# Supplementary material for: Analysis of German BSE Surveillance Data: Estimation of the Prevalence of Confirmed Cases versus the Number of Infected, but Non-Detected, Cattle to Assess Confidence in Freedom from Infection
Source: Int J Environ Res Public Health. 2021 Sep 22;18(19):9966. doi: 10.3390/ijerph18199966 (PMC8508286; doi:10.3390/ijerph18199966)
Supplement: Supplementary file 1 [file ijerph-18-09966-s001.zip › ijerph-1343507-supplementary.pdf]

International Journal of
Environmental Research and Public Health

MDPI

Supplementary Material

1. Introduction

We developed a Bayesian model for deriving three key outcomes from an analysis of German BSE surveillance data from 1990 to 2020 as described in Int. J. Environ. Res. Public Health (referred to as ``the paper''). The outcomes are i) estimation of birth cohort specific BSE prevalence, ii) estimates of the confidence in freedom from BSE for apparently free birth cohorts and iii) number of BSE-infected cattle that may have entered the food chain (``non-detects''). We provide here supplementary information about the priors, further modelling aspects, data and model used in the analysis as well as supplementary results.

2. Modelling aspects

Due to the low prevalence of BSE, a Poisson approximation was chosen for the expression in eq. (2) in the paper. Using the Poisson approximation, the second line of eq. (3) in the paper can be given as

$$C_{ij} = 1 - exp\left(-n_{ij}\,\pi'\,\delta\left(a_{ij}\right)\right) \tag{1}$$

and the "confidence" in freedom from BSE in the i-th birth cohort simplifies to

$$\begin{aligned} C_i &= 1 - Pr\left(X'_{i.} = 0 \,\middle|\, n_{i.},\ \pi',\ \delta\left(a_{ij}\right)\right) \\ &= 1 - exp\left[-\pi' \sum_j n_{ij}\ \delta\left(a_{ij}\right)\right] \end{aligned} \tag{2}$$

where X'_i is the total expected number of detected cases in cohort i out of $n_{i.} = \sum_j n_{ij}$ tested cattle under assumption of the design prevalence π'.

3. Priors

Our model requires prior information about the birth cohort-specific BSE prevalences π_i, for which we chose flat $beta(1{,}1)$ distributions. Furthermore, prior information about the parameters β_1, β_2 of the Gompertz function is required, which allows capturing of available knowledge and uncertainty about the age-dependent detection probability δ of BSE infection. For the purpose of sensitivity analysis, we induced different sets of priors from independent sources of information. The detection probability has been referred to as ``age-specific sensitivity'' [1], indicating that this probability is conditional on the true presence of infection.

3.1. Priors for Gompertz parameters: set 1

Our assumptions regarding the age-dependent detection probability $\delta(a)$, where a represents the age in years, are such that we expect values close to zero or close to 1.0 for very young or old cattle, respectively, where age is a proxy of the time since infection. This presupposes that infection occurs in the first year of life as described in the main paper. We assign a low level of uncertainty in specifying the expected detection probabilities at these extreme ages.

Furthermore, we assume that there is some unknown age, at which the expected detection probability δ has a maximum slope. Conceptually, BSE-infected cattle of this critical age would have the largest variability of being detected or non-detected. We also presuppose that the two-parameter Gompertz function is a suitable model to accommodate the shape of the unknown δ function. Our approach in deriving Gompertz priors is to generate data that reflect exactly these assumptions and to use these data for fitting the Gompertz model.

To this end, we consider the hypothetical data $\widehat{p_1} = 0/100$ and $\widehat{p_2} = 100/100$, which corresponds to expected results for estimating the age-dependent detection probability, with a relatively large sample size of 100 for the extreme age groups $x_1 = 0$ and $x_2 = 15$, respectively. Furthermore, we denote x_{crit} as the unknown critical age at which d is most uncertain with a prior uniform distribution $x_{crit} \sim U(1,7)$. For this critical age, we presuppose the hypothetical data $\widehat{p_{crit}} = 1/2$, reflecting high uncertainty. The R/rjags code for the model is

```
gom.set1.model <- function() {
 # priors
 b1 ~ dunif(-500,0)
 b2 ~ dunif(-10,0)
 x.crit ~ dunif(1,7)
 # response for critical age
 p.crit <- exp(b1 * exp(b2 * x.crit))
 y.crit ~ dbin(p.crit,s.crit)
 # responses for extreme age
 for(i in 1:2) {
  p[i]<- exp(b1 * exp(b2 * x[i]))
  y[i] ~ dbin(p[i],s[i])
 }
}
```

and uses the plausible ranges $[-500,0]$ and $[-10,0]$ as priors for the two required Gompertz parameters b1 and b2, respectively. Fitting the model to the hypothetical set 1 data

```
set1.data <- list(x=c(0,15),y=c(0,100),s=c(100,100,),y.crit=1,s.crit=2)
```

follows the same procedure as described in the main paper. For visualisation, we plotted a family of Gompertz functions using 250 random draws from the posterior joint distribution (Figure S1, upper left), confirming that the variety of curve shapes are in accordance with our understanding of the uncertainty about the age-dependent detection probability. Using the R package rriskDistributions [2], we selected the uniform and Normal distributions that best fit to 3000 random numbers from each of the posterior distributions of b1 and the transform log -b2 (not shown). The fitted posterior distributions represent set 1 priors for use in the main model (Table A1) and reflect the prior uncertainty about these parameters.

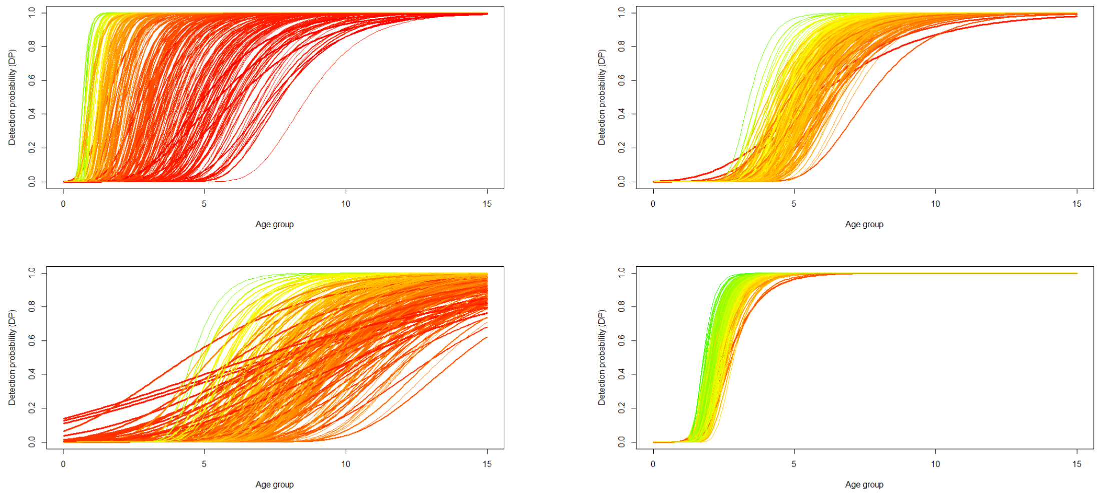

Figure S1: Visualisation of the Gompertz priors set 1 (upper left), 2a (upper right), 2b (lower left) and 3 (lower right) as Gompertz function plots using 250 pairs of parameters (β_1, β_2) chosen at random from the respective joint posterior distributions (see details in Priors). The line width and color differentiate the curves according to the parameter β_1 and β_2, respectively.

3.2. Priors for Gompertz parameters: set 2

[1] derived point estimates of the age-dependent detection probability (denoted α_a in Tab. 1 of the cited paper) by convolution of the probability density functions for the BSE susceptibility and incubation period, both empirically established based on the British BSE epidemic by [3]. An alternative quantification using the same source information accounts for the probability of survival without clinically apparent BSE up to the specified age group (denoted α'_a in Tab. 1 of the cited paper). We note here that the unconditional estimates (α_a) are more consistent with our assumption of a monotonous increase of d with age.

We use estimates of $\hat{p_i} = \alpha_a$ for fitting a Gompertz model, using the age group as independent variable x, with the simplifying assumption of normal errors. The R/rjags code for the model is

```
gom.set2.model <- function() {
 # priors
 b1 ~ dunif(-500,0)
 b2 ~ dunif(-10,0)
 tau ~ dgamma(1,1)
 for(i in 1:n) {
   p[i] ~ dnorm(mu[i], tau)
   mu[i] <- exp(b1 * exp(b2 * x[i]))
 }
}
```

using the same priors for b1 and b2 as with set 1 analysis and flat priors for the error term. The further procedure of deriving a distribution for the posterior distribution is as described for set 1 analysis. The fitted posterior distributions using the two vectors of point estimates α_a and α'_a represent sets 2a and 2b, respectively, for use as Gompertz priors in the main model Table S1). The uncertainty of the Gompertz parameters according to the analysis of sets 2a and 2b is visualised in Figure S1, upper right and lower left.

Table S1. Set of priors for the parameters of a Gompertz function modelling the age-dependent detection probability of BSE infection and their effect on BSE prevalence estimates.

Parameters	Set 1	Set 2a	Set 2b	Set 3
Priors[a]				
β_1	$U(-0.5, -9.7)$	$U(-503, -20.8)$	$U(-452.7, 79.7)$	$U(-520, -71.7)$
β_2	$N(0.8, 2.65)$	$N(0.07, 24.27)$	$N(-0.6, 6.76)$	$N(0.97, 42.96)$
Posteriors[b]				
β_1	-417.7 (496.2, -218.6)	-366.7 (-495.7, -153.2)	-360.1 (-448.7, -164.2)	-463.4 (-517.2, -303.1)
β_2	0.567 (0.452, 0.657)	0.534 (0.375, 0.628)	0.534 (0.393, 0.628)	0.606 (0.516, 0.690)
π_{2009}	2.8 (0.1, 14.1)	2.9 (0.1, 15.5)	2.8 (0.1, 15.4)	2.6 (0.1, 13.9)
π_{1996}	16.5 (13.9, 19,3)	16.6 (14.0, 19.4)	16.6 (14.0, 19.4)	16.4 (13.9, 19.2)
π_{1999}	5.6 (4.1, 7.2)	5.7 (4.3, 7.3)	5.6 (4.3, 7.4)	5.4 (4.2, 7.1)

[a] Uniform $\mathbf{U}(\text{min}, \text{max})$ and Normal $\mathbf{N}(\mu, \tau)$ distributions were fitted to posterior distribution of $\widehat{\beta_1}$ and $\log(-\widehat{\beta_2})$ respectively, obtained from precursor models (see section 0).[b] Median and 95 % credible interval of posterior distribution for the Gompertz parameters and three cohort-specific prevalence estimates (cases per 100,000 animals) for which the Gompertz parameter priors had the strongest effect.

3.3. Priors for Gompertz parameters: set 3

The German BSE pathogenesis study provided data on the detectability of the BSE agent in the obex, which is also the testing organ for surveillance. The details of the experimental setting are described by [4]. Briefly, a total of 56 calves between 4 and 6 months of age were orally challenged with a brain-stem homogenate pool of clinically diseased cattle (total dose of $10^{8.1}$ ID_{50} per animal). The occurrence of the BSE agent was investigated in several tissues using immunohistochemistry. For deriving Gompertz priors, only the detection of BSE agent in the obex is of interest, because this setting resembles most closely the conditions of BSE surveillance. Results by [5], which were available for analysis, are summarised in Table S2.

Table S2. Results of the German BSE pathogenesis study extracted from [5].

n	Month p.i.	Age group [a]	ObexIHC [b]
28	16	1	0
46	16	1	0
50	20	2	0
60	20	2	0
24	24	2	0
58	24	2	0
21	28	2	1
52	28	2	0
9	32	3	1
61	32	3	1
11	36	3	1
49	36	3	1
25	49	3	1
56	40	3	1
22	44	4	1
38	44	4	1

p.i. = *post infection.* [a] BSE model age group with a midpoint closest to age at testing (established as months *p.i.* plus five to account for mean age at challenge). [b] Obex immunohistochemistry: 0 = neg, 1 = pos.

As individual age at testing, we used the reported month p.i. and added five months to account for the mean age at challenge. The animals were then allocated to an age group (as defined above) by choosing the age group with a midpoint closest to the age at testing. For example, animal 28 has been tested at age of about 21 months, which is closest to age group 1 with a midpoint of 18 months. Let x_i, D_i and n_i denote the age group in years at testing, number of detected cattle and number of cattle tested per age group, respectively, with index $i = 1, \dots, n$ referring to the given age group in Table S3. The precursor model of the observed binomial outcome D_i uses the Gompertz function to derive an age-dependent probability p_i,

$$\begin{aligned} D_i &\sim bin(p_i, n_i), \\ p_i &= exp(\beta_1\ exp(\beta_2\ x_i)). \end{aligned} \quad (3)$$

The R/rjags code for the model is

```
gom.set3.model <- function() {
 # priors
 b1 ~ dunif(-500,0)
 b2 ~ dunif(-10,0)
 for(i in 1:N) {
   D[i] ~ dbin(p[i], n[i])
   p[i] <- exp(b1 * exp(b2 * x[i]))
 }
}
```

Priors for b1 and b2, and further procedures are as described for set 1 analysis. The resulting distributions for set 3 for use as priors in the main model are given in Table S1 and the uncertainty of the Gompertz function is visualised in Figure S1, lower right.

Table S3. Age group, number of detected cattle and number of cattle tested per age group in the German pathogenesis study [5], used for inducing set 3 of Gompertz priors.

Age group[a] (x)	Number of detects (D)	Number tested (n)
1	0	2
2	1	6
3	6	6
4	2	23

[a] Age group for BSE model (see Table A2).

4. Model verification and sensitivity analysis

The Gompertz function was selected as model for the latent age-dependent detection probability because it captured the assumed properties and provided sufficient flexibility for its shape to accommodate various sources of information. It was also chosen because it provided the best fit (Akaike's information criterion; Cademo software, WACH-Version 3.17, BioMath GmbH, Germany) to the derived estimates α_a (see section 0) compared with nine alternative functions including exponential, logistic, and the Bertalanffy and Janoschek functions (results not shown).

For the purpose of model verification, we generated a synthetic data set of the response variable X_{ij} using the Poisson-Gompertz model with (assumed) known parameters. Using these simulated data, we obtained the cohort-specific

estimates for prevalence and compared them with the true values. The results were unbiased and more precise for birth cohorts with larger available sample sizes (results not shown). This reflects correct statistical inference.

We analysed the uncertainty due to the choice of Gompertz priors (set 1, 2a, 2b and 3) covering all available independent sources of information about the age-dependent detection probability (δ). The posterior estimates of the Gomperz parameters do not deviate markedly among the different sets of priors Table S1. The size of the effect is shown in terms of the posterior expected δ (Figure S2, top). To evaluate the effect of the Gompertz priors on our key outcome, which is the birth-cohort specific prevalences, we identified those birth cohorts for which the greatest deviations were observed (Table S1 and Figure S2, bottom). The effect on the key outcome appears to be relatively mild. Final inference was based on set 1 Gompertz priors.

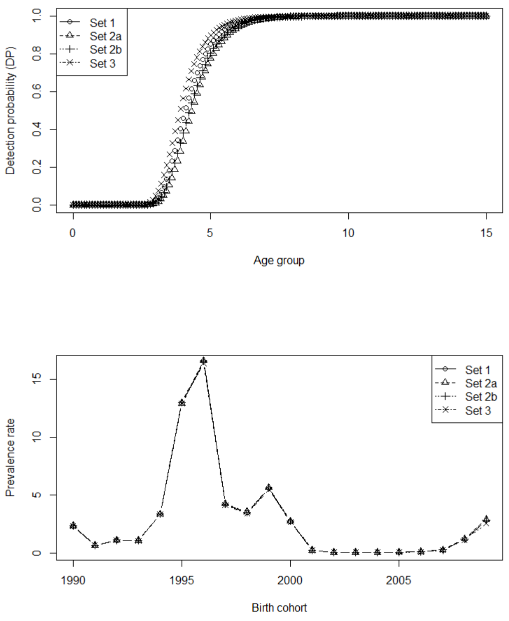

Figure S2. Posterior estimates of the Gompertz function for expressing the detection probability (*δ*) (top) and cohort-specific BSE prevalences (cases per 100,000 animals) (bottom) based on four different sets of priors for the Gompertz parameters (see text for further details).

5. Main model

The main model in R/rjags syntax is

```
jags.model <- function() {
 b1 ~ dunif(prior.b1[1],prior.b1[2]) # prior for Gompertz parameter
 b2 ~ dnorm(prior.b2[1],prior.b2[2]) # prior for Gompertz parameter
 for(i in 1:B) {                  # index for birth cohort (rows)
  pi[i] ~ dbeta(1,1)              # prior for prevalences
  R[i]  <- pi[i]*1.0e5            # prevalence rate (cases/100,000)
  # confidence for freedom for cohort (only for cohorts without cases)
  C[i] <- (1.0 - prod(pneg[i,i:T])) * (sum(x[i,i:T]) == 0)
  for(j in i:T ) {               # index for testing year (columns)
   # Gompertz function for age (a) dependent detection probability (delta or Se)
   Se[i,j] <- exp(b1 * exp(-exp(b2) * a[i,j]))
   lambda[i,j] <- n[i,j] * pi[i] * Se[i,j]
   # P(all Tests negative at design prev 1/100,000)
```

```
      pneg[i,j] <- (1 - 1.0e-5 *Se[i,j])**n[i,j]
      x[i,j] ~ dpois(lambda[i,j])
      nd[i,j] <- 1.0e5*pi[i]*(1-Se[i,j]) # fixed rate per 100,000 slaughters
    }
  }
  for(j in 1:T){
    D[j] <- sum(nd[1:j,j])/j # rescaling to annual total number of cases/ 100,000
  }
}
```

6. Data

The analysis of the German BSE surveillance data was based on data collected by the Friedrich-Loeffler Institute (FLI). The number of BSE cases detected and the number of tests conducted from 1990 to 2013 are given in Table S4 and Table S5, respectively. Only cells of the data matrices with non-missing number of detected cases (values equal or greater zero in Table 5) were used for the model. Note that sample sizes of ``1'' in Table S5 were actually used in the model in three situations, namely for animals from cohorts 1991, 1997 and 1998, which were tested in the years 1998, 1997 and 1998, respectively.

Table S4. German BSE surveillance data 1990--2013: Number of BSE cases detected per year of testing (columns) and birth cohort (rows).

Cohort	1990	1991	1992	1993	1994	1995	1996	1997	1998	1999	2000	2001	2002	2003	2004	2005	2006	2007	2008	2009	2010	2011	2012	2013
1990										0	0	1	1	0										
1991									0	0	0	1	0	0	1									
1992										0	0	1	1	0	0	0								
1993										0	0	0	3	0	0	0	0							
1994										0	1	7	5	0	2	0	0	0						
1995										0	2	39	31	8	2	1	1	0	0					
1996										0	4	69	46	12	9	3	0	0	0	1				
1997								0		0	0	5	10	13	13	1	0	0	0	0	0			
1998									0	0	0	2	8	8	10	5	1	0	0	0	0	0		
1999										0	0	0	0	13	18	12	9	3	0	0	0	0	0	
2000											0	0	0	0	10	8	5	1	2	1	0	0	0	0
2001													0	0	0	2	0	0	0	0	0	0	0	0
2002														0	0	0	0	0	0	0	0	0	0	0
2003															0	0	0	0	0	0	0	0	0	0
2004																0	0	0	0	0	0	0	0	0
2005																	0	0	0	0	0	0	0	0
2006																		0	0	0	0	0	0	0
2007																			0	0	0	0	0	0
2008																				0	0	0	0	0
2009																					0	0	0	0
2010																						0	0	0
2011																							0	0
2012																								0

Table S5. German BSE surveillance data 1990--2013: Number of BSE tests conducted per year of testing (columns) and birth cohort (rows).

Cohort	1990	1991	1992	1993	1994	1995	1996	1997	1998	1999	2000	2001	2002	2003	2004	2005	2006	2007	2008	2009	2010	2011	2012	2013
1990	1	1	1	1	1	1	1	1	1	13	38	47378	12626	53516	1	1	1	1	1	1	1	1	1	1
1991		1	1	1	1	1	1	1	1	18	51	67478	17870	19254	297931	1	1	1	1	1	1	1	1	1
1992			1	1	1	1	1	1	1	29	111	97125	25451	27251	71153	22497	1	1	1	1	1	1	1	1
1993				1	1	1	1	1	1	43	192	138255	36634	38813	84720	16624	23452	1	1	1	1	1	1	1
1994					1	1	1	1	1	57	238	192702	52147	55865	97691	26603	15968	23712	1	1	1	1	1	1
1995						1	1	1	1	76	304	255110	96058	79523	113119	43422	25929	15441	26571	1	1	1	1	1
1996							1	1	1	98	306	318827	130782	144588	126418	67829	42160	25317	17224	24128	1	1	1	1
1997								1	1	168	477	368972	167468	193303	141693	102088	65404	39666	26111	14085	23911	1	1	1
1998									1	248	1030	330974	205855	247164	154058	147653	98715	61686	41247	24323	14808	23673	1	1
1999										315	719	411192	236234	294432	169112	201343	143490	92827	64022	39182	23812	14598	20575	1
2000											567	426492	1230752	330597	177137	261041	201388	138919	100576	64597	39160	24288	12979	20136
2001												1	771684	586291	193372	312013	261374	194754	150277	100944	64166	40359	21942	12638
2002													1	511221	532916	334608	295776	239254	199219	144323	95788	62508	34636	20729
2003														1	370828	481405	320545	276604	248589	196165	138725	95996	55157	33006
2004															1	57261	365336	302994	295653	254019	196331	147326	89938	55462
2005																1	28280	229024	319728	301649	254378	210081	135451	88658
2006																	1	10162	226591	16751	292412	160421	187634	78150
2007																		1	7685	13373	7492	181342	34287	107492
2008																			1	3366	8174	4491	37417	33708
2009																				1	36209	7651	1762	35668
2010																					1	36490	1632	1694
2011																						1	1396	1636
2012																							1	1136
2013																								1

7. Results

The birth cohort-specific prevalences are given in Table S6. For birth cohorts 2010–2013, the estimates converge to the prior prevalence (50~\% or 50,000 cases out of 100,000 animals tested) due to the lack of data (not shown). See main paper for further results.

Table S6. Model estimates of BSE infection prevalence (cases per 100,000 animals) in the German cattle population.

Birth cohort	Prevalence (95 % credible interval)
1990	2.297 (0.559, 6.368)
1991	0.657 (0.154, 1.771)
1992	1.084 (0.247, 3.071)
1993	1.068 (0.340, 2.625)
1994	3.377 (1.964, 5.343)
1995	12.976 (10.361, 15.873)
1996	16.502 (13.918, 19.345)
1997	4.155 (3.086, 5.584)
1998	3.504 (2.473, 4.784)
1999	5.582 (4.210, 7.226)
2000	2.721 (1.814, 3.968)
2001	0.250 (0.053, 0.666)
2002	0.064 (0.002, 0.353)
2003	0.070 (0.002, 0.383)
2004	0.071 (0.002, 0.395)
2005	0.077 (0.002, 0.410)
2006	0.113 (0.004, 0.582)
2007	0.265 (0.009, 1.353)
2008	1.124 (0.039, 6.053)
2009	2.834 (0.108, 14.694)

References

1. Böhning, D. and Greiner, M., Evaluation of the cumulative evidence for freedom from BSE in birth cohorts. Eur.J.Epidemiol., **2006**, 21(1):47 54.
2. Belgorodski, N., Greiner, M., Tolksdorf, K., and Schueller, K., rriskDistributions: Fitting distributions to given data or known quantiles. R package version 1.8., **2012.**
3. Ferguson, N., Donnelly, C., Woolhouse, M., and Anderson, M., The epidemiology of BSE in cattle herds in Great Britain. II. Model construction and analysis of transmission dynamics. Philos.Tr.R.Soc.London, **1997**, 352:803 838.
4. Hoffmann, C., Ziegler, U., Buschmann, A., Weber, A., Kupfer, L., Oelschlegel, A., Hammerschmidt, B., and Groschup, M. H. Prions spread via the autonomic nervous system from the gut to the central nervous system in cattle incubating bovine spongiform encephalopathy. J Gen Virol, **2007**, 88(Pt 3):1048 1055.
5. Kaatz, M., Fast, C., Ziegler, U., Balkema-Buschmann, A., Hammerschmidt, B., Keller, M., Oelschlegel, A., McIntyre, L., and Groschup, M. H., Spread of classic BSE prions from the gut via the peripheral nervous system to the brain. Am J Pathol, **2012**, 181(2):515 524.
